# Dual Mechanisms of Cardiac Action Potential Prolongation by 4-Oxo-Nonenal Increasing the Risk of Arrhythmia; Late Na^+^ Current Induction and hERG K^+^ Channel Inhibition

**DOI:** 10.3390/antiox10071139

**Published:** 2021-07-19

**Authors:** Seong-Woo Choi, Ming-Zhe Yin, Na-Kyeong Park, Joo-Han Woo, Sung-Joon Kim

**Affiliations:** 1 Department of Physiology, Dongguk University College of Medicine, Gyeongju 38066, Korea; physiolcsw@dongguk.ac.kr (S.-W.C.); gabriel929@dongguk.ac.kr (J.-H.W.); 2 Department of Physiology, Seoul National University College of Medicine, Seoul 03080, Korea; myungchul5_5@snu.ac.kr (M.-Z.Y.); pnk96@snu.ac.kr (N.-K.P.); 3 Department of Anesthesiology, Second Affiliated Hospital of Zhejiang University School of Medicine, Hangzhou 310058, China

**Keywords:** lipid peroxidation, 4-oxo-nonenal, heart, arrhythmia, late Na^+^ current

## Abstract

4-Oxo-nonenal (4-ONE) is an endogenous lipid peroxidation product that is more reactive than 4-hydroxy-nonenal (4-HNE). We previously reported the arrhythmic potential of 4-HNE by suppression of cardiac human Ether-a-go-go Related Gene (hERG) K^+^ channels with prolonged action potential duration (APD) in cardiomyocytes. Here, we illustrate the higher arrhythmic risk of 4-ONE by modulating the cardiac hNa_V_1.5 channel currents (I_NaV_). Although the peak amplitude of I_NaV_ was not significantly changed by 4-ONE up to 10 μM, the rate of I_NaV_ inactivation was slowed, and the late Na^+^ current (I_NaL_) became larger by 10 μM 4-ONE. The chemical modification of specific residues in hNa_V_1.5 by 4-ONE was identified using MS-fingerprinting analysis. In addition to the changes in I_NaV_, 4-ONE decreased the delayed rectifier K^+^ channel currents including the hERG current. The L-type Ca^2+^ channel current was decreased, whereas its inactivation was slowed by 4-ONE. The APD prolongation by 10 μM of 4-ONE was more prominent than that by 100 μM of 4-HNE. In the computational in silico cardiomyocyte simulation analysis, the changes of I_NaL_ by 4-ONE significantly exacerbated the risk of arrhythmia exhibited by the TdP marker, qNet. Our study suggests an arrhythmogenic effect of 4-ONE on cardiac ion channels, especially hNa_V_1.5.

## 1. Introduction

Reactive carbonyl species (RCS), such as 4-hydroxy-nonenal (4-HNE) and 4-oxo-nonenal (4-ONE), are secondary peroxidation products of unsaturated fatty acids [1,2]. The ability of RCS to covalently react with the nucleophilic groups of nucleic acids and proteins exerts various pathophysiological consequences [3,4,5,6,7,8]. The heart is vulnerable to reactive oxygen species (ROS) and RCS produced by oxidative damage in ischemia/reperfusion, fibrillation, and heart failure [9,10,11,12]. Despite the importance of altered ion channel functions in cardiac diseases, the pathophysiological plausibility of interactions between ion channels and RCS has rarely been investigated.

We previously reported that 4-HNE has a potential arrhythmic effect on the heart by extending the action potential duration (APD), which was mediated by the inhibition of human Ether-a-go-go Related Gene (hERG) K^+^ channel current (I_Kr_) [13]. In addition to the voltage-gated K^+^ channels, such as hERG, various functional disturbances of the human cardiac Na^+^ channel (hNa_V_1.5) are associated with an increased risk of arrhythmia [14]. The *SCN5A* gene encodes the hNa_V_1.5 α-subunit, and mutations in *SCN5A* are associated with inherited susceptibility to ventricular arrhythmia, such as Brugada syndrome, long QT syndrome class 3 (LQT-3), or atrial fibrillation [15,16].

A gain-of-function mutation of *SCN5A* leads to increased Na^+^ influx during systole, resulting in delayed action potential repolarization or early afterdepolarization (EAD) of the cardiac AP [16]. Specifically, the persistent or non-inactivating component of hNa_V_1.5, called the late Na^+^ current (I_NaL_), could be responsible for the prolonged APD of LQT-3. However, the chemical modification of hNa_V_1.5 and its arrhythmogenic effect, such as I_NaL_ induction, has been rarely investigated. Interestingly, previous studies have shown that the oxidative condition of cardiac ischemia and heart failure enhanced I_NaL_ [17,18,19]. The plausible changes of hNa_V_1.5 current (I_NaV_) and the putative induction of I_NaL_ in ROS-mediated arrhythmia attracted us to investigate the modification of hNa_V_1.5 activity by RCS.

In the present study, we highlighted the arrhythmic potentials of 4-ONE, which is formed from 4-hydroperoxy-2-nonenal, the same precursor as 4-HNE [1]. Structurally, 4-ONE differs at the C4 position with a ketone group instead of the hydroxyl group of 4-HNE, increasing the electrophilic reactivity of 4-ONE. Therefore, 4-ONE modifies various nucleophilic amino acids, such as cysteine (Cys), lysine (Lys), histidine (His), and arginine (Arg) [20,21,22]. However, a previous study on the effects of 4-ONE on ion channel activity was limited to TRPA1 and TRPV1 nonselective cation channels as the harmful sensory signals [23]. In addition to hNa_V_1.5, we also examined the effects of 4-ONE on hERG (I_Kr_), *KCNQ1/KCNE1* (I_Ks_), and L-type voltage-operated Ca^2+^ channels (I_Ca,L_). Finally, the relative contribution of the I_NaV_ modulation to APD prolongation and arrhythmogenic risk was analyzed by a recently announced method of proarrhythmic risk analysis called Comprehensive in vitro Proarrhythmia Assay (CiPA), cooperatively using experimental data and in silico simulation [24,25].

## 2. Materials and Methods

### 2.1. Cell Preparation

HEK-293 cell line cells stably overexpressing hNa_V_1.5 (hNa_V_1.5-HEK cell) or hERG1a (hERG-HEK cell) were used for the electrophysiological recording of I_NaV_ and I_Kr_, respectively. The hNa_V_1.5-HEK cell was kindly donated by Dr. Jae-Hong Ko (Chung-Ang University, Seoul, Korea). The hNa_V_1.5-HEK cells were maintained in DMEM (Thermo Fisher Scientific, Bremen, Germany) supplemented with 10% FBS (Serana Europe, Pessin, Germany) and geneticin G418 (Sigma-Aldrich, Saint Louis, MO, USA). The hERG-HEK cell was kindly donated by Dr. Han Choe (University of Ulsan, Seoul, Korea). The hERG-HEK cells were maintained in MEM (Thermo Fisher Scientific) supplemented with 10% FBS. To record the slowly activating voltage-dependent K^+^ current (I_Ks_), HEK cells were transiently overexpressed with *KCNQ1* and *KCNE1* plasmid DNA (RG219869 and RC225088, OriGene Technologies, Rockville, MD, USA) using FuGENE 6 kit (Roche, Penzberg, Germany). To record L-type Ca^2+^ current (I_Ca,L_) and cardiac action potential (AP), guinea-pig ventricular myocytes (GPVMs) were isolated using the Langendorff apparatus as described previously [13].

### 2.2. Electrophysiological Recording

Conventional whole-cell voltage and current-clamp were conducted for currents and AP recordings, respectively. For the I_NaV_ recording, high giga-seal resistance (>2 GΩ), low series resistance (<10 MΩ), and the series resistance compensation (80%) were introduced to reduce voltage-clamp error. The extracellular bath solution for the I_NaV_ and I_NaL_ recordings in hNa_V_1.5-HEK cells contained 130 mM NaCl, 10 mM HEPES (4-(2-hydroxyethyl)-1-piperazineethanesulfonic acid), 4 mM CsCl, 1 mM MgCl_2_, 2 mM CaCl_2_, and 10 mM glucose adjusted to pH 7.4 with NaOH. The intracellular pipette solution for the I_NaV_ and I_NaL_ recordings contained 117 mM CsCl, 20 mM NaCl, 1 mM MgCl_2_, 5 mM HEPES, 5 mM EGTA, 5 mM MgATP, and 0.4 mM TrisGTP adjusted to pH 7.3 with CsOH. The extracellular bath solution for the I_Kr_ and I_Ks_ recordings contained 145 mM NaCl, 3.6 mM KCl, 10 mM HEPES, 1 mM MgCl_2_, 1.3 mM CaCl_2_, and 5 mM glucose adjusted to pH 7.4 with NaOH. The intracellular pipette solution for the I_Kr_ and I_Ks_ recordings contained 100 mM K-aspartate, 25 mM KCl, 5 mM NaCl, 10 mM HEPES, 1 mM MgCl_2_, 4 mM MgATP, and 10 mM BAPTA adjusted to pH 7.25 with KOH. The extracellular bath solution for I_Ca,L_ contained 145 mM CsCl, 10 mM HEPES, 1 mM MgCl_2_, 1.8 mM CaCl_2_, and 5 mM glucose adjusted to pH 7.4 with CsOH. The intracellular pipette solution for I_Ca,L_ contained 106 mM CsCl, 20 mM TEA-Cl, 5 mM NaCl, 10 mM HEPES, 5 mM MgATP, and 10 mM EGTA adjusted to pH 7.25 with CsOH. The compositions of the extracellular solutions used for the AP recording contained 145 mM NaCl, 5.4 mM KCl, 10 mM HEPES, 1 mM MgCl_2_, 1.8 mM CaCl_2_, and 5 mM glucose adjusted to pH 7.4 with NaOH. The intracellular solution contained 120 mM K-aspartate, 20 mM KCl, 5 mM NaCl, 2 mM CaCl_2_, 5 mM EGTA, 10 mM HEPES, and 5 mM MgATP adjusted to pH 7.25 with KOH.

### 2.3. In Silico Simulation

CiPAORdv1.0 (modified O’Hara–Rudy model) was used to simulate human ventricular AP and its changes due to the altered ionic currents (I_Kr_, I_Ks_, I_Ca,L_, I_NaV_, and I_NaL_) by 4-ONE and 4-HNE. The levels of ionic current inhibition and the equations of inactivation time constant obtained from the experimental results are presented in Table 1.

### 2.4. Tandem Mass Spectrometry

The total lysates of the hNa_V_1.5-HEK cells treated with 4-ONE (10 μM) were subjected to SDS-PAGE for mass spectrometry (MS). The hNa_V_1.5 bands were cut from the SDS-PAGE gel and digested in gel with trypsin (Promega, Madison, WI, USA). The subsequent procedures were similar to the previous MS [13]. A fragment mass tolerance of 1.0 Da, peptide mass tolerance of 25 ppm, and maximum missed cleavage of 2 were set. The result filters were performed with charge states versus scores (XCorr by Sequest) where the minimal scores for the charge states were +1: 1.6, +2: 1.7, +3: 3.0, and >+4: 3.5. The carbamidomethylation (+57.021 Da) of cysteine (C) was set as a static modification, and the following variable modifications were allowed: Michael addition, +154 Da (C, H, K, R); Schiff base addition, +136 Da (C, H, K); and oxidation, +15.995 Da (M). The respective data for the post-translational modification (PTM) sites by 4-ONE were transformed and analyzed with Scaffold 4 program (Proteome Software, Portland, OR, USA).

### 2.5. Chemicals

The compounds 4-ONE and 4-HNE were purchased from Cayman Chemical (Ann Arbor, MI, USA). The 4-ONE and 4-HNE were stored in 20 mM stocks in DMSO at −20 °C. Immediately prior to the application to the cells, 4-ONE and 4-HNE were freshly diluted with extracellular bath solution to the final target concentrations. Application of 4-ONE and 4-HNE was processed for at least 5 min to obtain stable electrophysiological responses. Other chemicals were purchased from Sigma-Aldrich (St. Louis, MO, USA).

### 2.6. Statistical Analysis

Data are expressed as mean ± S.E., and the statistical analyses were determined using paired or unpaired Student’s *t*-tests. A *p*-value < 0.05 was considered as statistically significant.

## 3. Results

### 3.1. Slowed hNa_V_1.5 Inactivation and I_NaL_ Induction by 4-ONE

**Figure 1:**
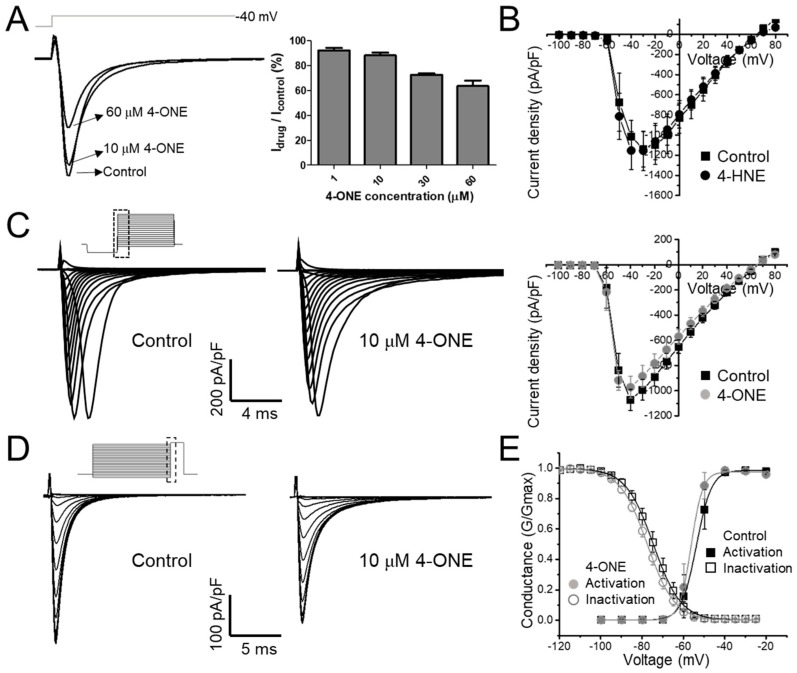
The modulations of hNa_V_1.5 channel by 4-oxo-nonenal (4-ONE) were evaluated in HEK-293 cell line cells stably overexpressing hNa_V_1.5 (hNa_V_1.5-HEK cell). (**A**) The Na_V_1.5 current (I_NaV_) was activated by applying a depolarization pulse to −40 mV from −120 mV of hyperpolarized potential. (**B**,**C**) The current–voltage (I–V) relationship was analyzed by applying multistep depolarization pulse protocol from −100 to 80 mV from −120 mV of hyperpolarization for 200 ms. (**B**) I–V relationship for control and 100 μM 4-hydroxy-nonenal (4-HNE)-treated I_NaV_. (**C**) Raw traces and I–V relationship of I_NaV_ for control and 10 μM 4-ONE. (**D**) Steady-state inactivation of I_NaV_ was analyzed at −20 mV by applying 200 ms of pre-inactivating voltages from −120 to −25 mV. (**E**) Relative conductance of inactivation and activation voltage dependences was analyzed by 10 μM 4-ONE applications.

The effect of 4-ONE on cardiac hNa_V_1.5 was evaluated using stably overexpressing hNa_V_1.5 in the HEK-293 cell line (hNa_V_1.5-HEK cell). In the whole-cell voltage-clamp condition, the inward I_NaV_ was recorded by applying −40 mV of depolarization pulse (300 ms) from −120 mV holding potential. After confirming the stable recording of I_NaV_, 4-ONE was applied to the bath perfusing solution, which reduced the peak amplitude of I_NaV_ in a dose-dependent manner (Figure 1A; remaining current after 4-ONE treatment: 94.25% for 1 μM, 88.22% for 10 μM, 72.53% for 30 μM, and 63.66% for 60 μM 4-ONE; *n* = 3, *n* = 6, *n* = 3, and *n* = 3;, respectively). The reduced I_NaV_ was not restored by washing 4-ONE (data not shown), which was similar to the irreversible effect of 4-HNE on I_hERG_, as previously reported [13]. It has been reported that the in vivo concentration of 4-HNE under pathophysiological conditions ranges 1–100 μM [1]. In contrast, the in vivo concentration of 4-ONE has not been reported. However, an experiment on EA.hy 926 endothelial cells treated with ferrous sulfate suggested that the endogenous concentration of 4-ONE could be increased to 20 μM [26]. Therefore, we applied 10 μM 4-ONE in the subsequent experiments.

The current–voltage (I–V) relationship curves of the I_NaV_ showed a minute decrease in the peak amplitude at 10 μM 4-ONE (Figure 1C; peak inward current at −40 mV of −1071.8 ± 84.17 and −972.3 ± 85.52 pA/pF for control and 4-ONE, respectively, *n* = 6), whereas 4-HNE had no significant effect even at 100 μM (Figure 1B). The I–V curves were converted to the conductance–voltage (G–V) curve for analyzing the voltage dependence of hNa_V_1.5, which showed a slight left-shift, indicating that 4-ONE could reduce the threshold of activation (Figure 1E; half-maximal voltage of activation of −53.7 and −56.3 mV for control and 4-ONE, respectively, *n* = 6). The steady-state inactivation property of hNa_V_1.5 was analyzed by using the double pulse protocol (Figure 1D, inset). The steady-state inactivation curve also showed a slight left-shift by 4-ONE (Figure 1D; half-maximal voltage of inactivation of −74.5 and −77.5 mV for control and 4-ONE, respectively, *n* = 6).

**Figure 2:**
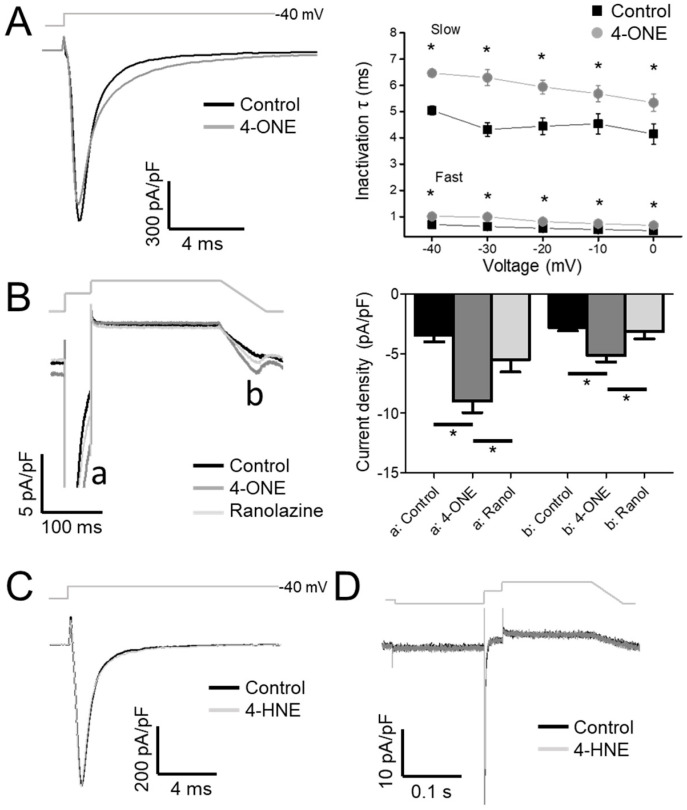
The inactivation decay of I_NaV_ and the late Na^+^ current (I_NaL_) by 4-ONE were analyzed. (**A**) I_NaV_ was activated by applying depolarization pulses (−40, −30, −20, −10, and 0 mV) from −120 mV of holding potential. The current decay was analyzed using double exponential fitting. (**B**) I_NaL_ through hNa_V_1.5 channel was recorded by applying action potential-like repolarization pulse protocol. The I_NaV_ was activated by short depolarization to −20 mV from −120 mV of hyperpolarized potential (a). The resurgent I_NaL_ was then recorded during ramp pulse repolarization (b). (**C**,**D**) 4-HNE treatment induced neither the inactivation decay of I_NaV_ nor the I_NaL_. All the data were analyzed using paired *t*-tests, where a *p* < 0.05 was considered statistically significant (*).

Upon analysis of the inactivation speed of I_NaV_, the rate of inactivation that was slowed by 4-ONE (Figure 2A, left) was notable. When the normalized decaying components of I_NaV_ were fit to a double exponential equation, both time constants for the fast and the slow components (τ_fast_ and τ_slow_) were increased by 4-ONE (Figure 2A, right). The delayed inactivation of I_NaV_ suggested an increase in I_NaL,_ which is the residual activity of hNa_V_1.5 that was flowing after the large peak Na^+^ current during AP. To analyze I_NaL_ more specifically, we applied the AP-like voltage-clamp protocol, two-step depolarization followed by a reverse-ramp voltage pulse (Figure 2B, upper gray line). The resurgent inward current during the reverse-ramp period reflected the augmented I_NaL_ by 4-ONE (Figure 2B, b; current density of −2.79 ± 0.27 and −5.16 ± 0.53 pA/pF for control and 4-ONE, respectively, *n* = 13). The sustained current at 50 ms after the peak Na^+^ influx was increased as well (Figure 2B, a; current density of −3.44 ± 0.56 and −8.99 ± 0.96 pA/pF for control and 4-ONE, respectively, *n* = 13). The increased inward currents (a and b) in the presence of 4-ONE were reversed by additional application of 50 μM of ranolazine, a late Na^+^ current inhibitor (Figure 2B, −5.50 ± 1.04 and −3.14 ± 0.60 pA/pF, a and b, respectively, *n* = 6). In contrast to the significant induction of I_NaL_ by 10 μM 4-ONE, the application of 100 μM 4-HNE induced neither slower inactivation nor I_NaL_ (Figure 2C,D).

**Figure 3:**
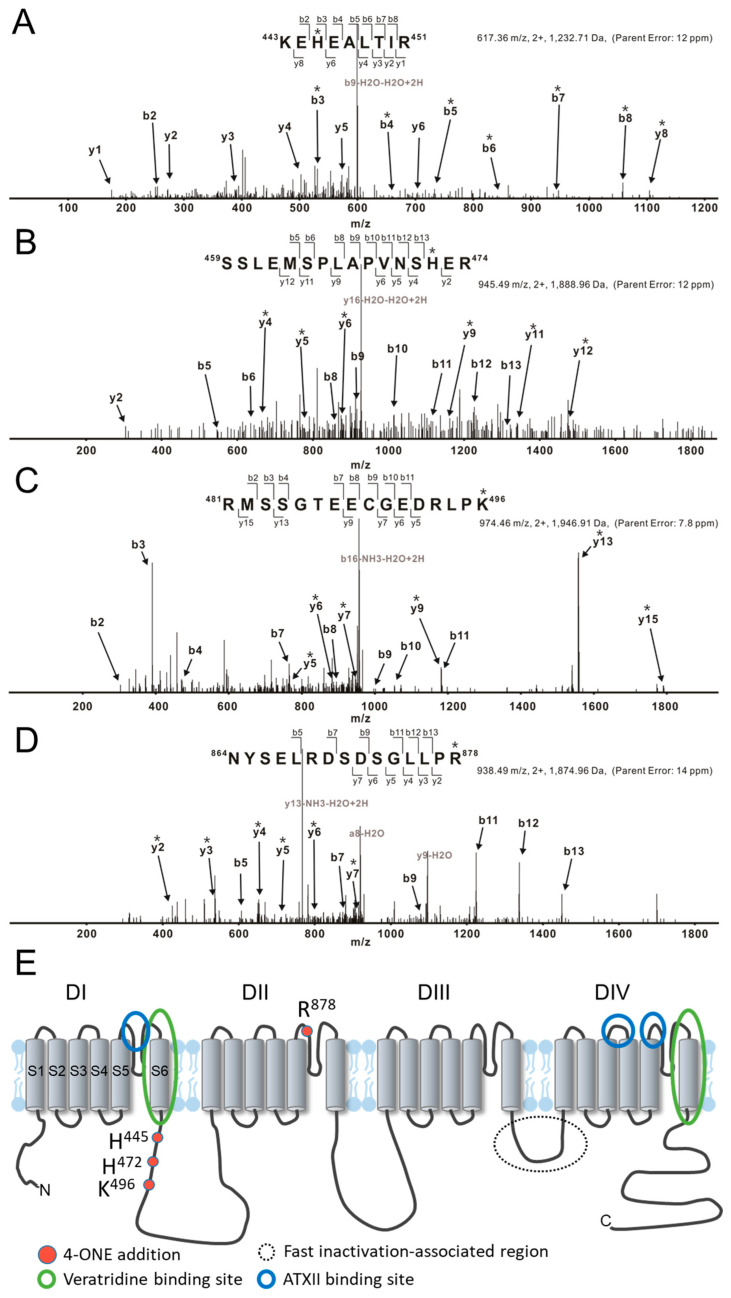
LC/MS/MS CID mass spectra of 4-ONE-modified hNa_V_1.5 peptides. (**A**–**D**) Tryptically digested peptides are fragmented to KEhEALTIR, SSLEMSPLAPVNShER, RmSSGTEECGEDRLPk, and NYSELRDSDSGLLPr, respectively. The sites of the 4-ONE Schiff base addition (**A**–**C**) or Michael addition (**D**) are localized to His^445^, His^472^, Lys^496^, and Arg^878^ by analysis of b and y ion fragmentation patterns. The product ions containing 4-ONE addition are indicated with asterisks (*). (**A**) The mass addition of 136 to the b and y ions containing His^445^, including y8 and b3–b8, combined with the absence of this addition to y1–y4, y6, and b2 identifies His^445^ as the 4-ONE-modified amino acid. (**B**) Ions b5, b6, b8–b13, and y2 lack the addition of 136 Da that is present on ions y4–y6, y9, y11, and y12, thus localizing the Schiff base adduct to His^472^. (**C**) Ions b2–b4 and b7–b11 lack the addition of 136 Da that is present on ions y5–y7, y9, y13, and y15 (Lys^496^). (**D**) Ions b5, b7, b9, and b11–b13 lack the addition of 154 Da and ions containing Arg^878^ show the addition (y2–y7). (**E**) The topological structure of hNa_V_1.5, including 4-ONE addition amino acids and binding sites. The previously known binding regions of I_NaL_ activators veratridine and *Anemonia viridis* toxin 2 are marked with blue and green circles, respectively.

The effects of 4-ONE on I_NaV_ could be due to the PTM of hNa_V_1.5, i.e., direct binding of 4-ONE with nucleophilic amino acids, such as Cys, His, Lys, and Arg [20,27]. The tandem MS of hNa_V_1.5 with or without 4-ONE treatment revealed four different sites of modification: His^445^, His^472^, Lys^496^, and Arg^878^. The representative MS/MS spectrum for the peptides ^443^KEhEALTIR^451^, ^459^SSLEMSPLAPVNShER^474^, and ^481^RmSSGTEECGEDRLPk^496^ shows that His^445^, His^472^, and Lys^496^ were commonly modified with Schiff base addition (Figure 3A–C). Another spectrum shows the Michael addition of Arg^878^ in the peptide ^864^NYSELRDSDSGLLPr^878^ (Figure 3D). The 4-ONE-binding sites were visualized with the schematic topology of the hNa_V_1.5 channel (Figure 3E). The sites of the Schiff base addition were located at the intracellular linker between domain I (DI) and domain II (DII). The site of the Michael addition was located at the extracellular S5–S6 linker of DII.

### 3.2. Multiple Effects of 4-ONE on I_Kr_, I_Ks_, and I_Ca,L_

**Figure 4:**
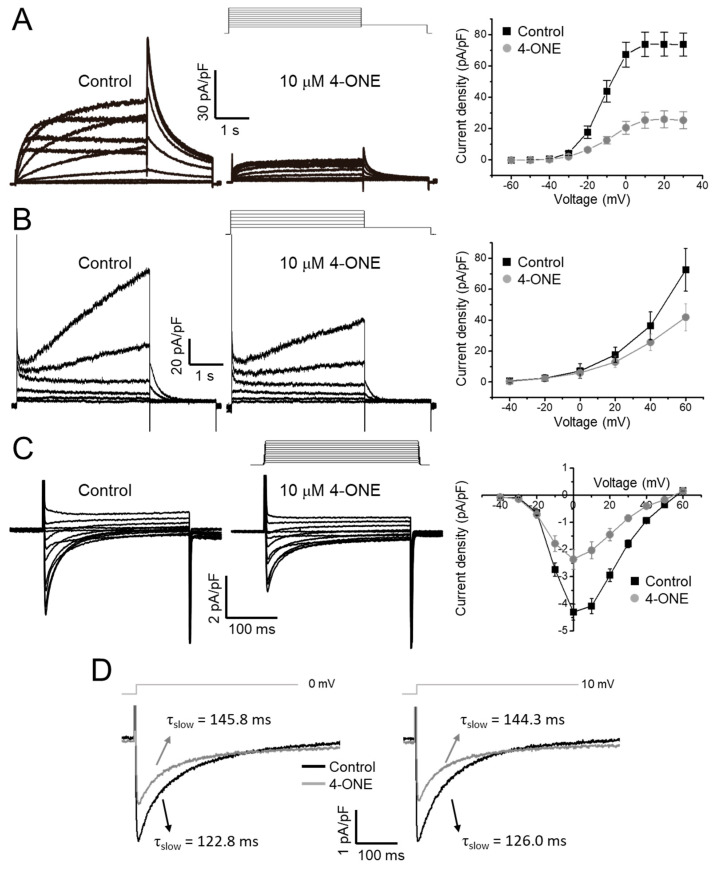
Effects of 4-ONE on cardiac ionic currents. (**A**) Human Ether-a-go-go Related Gene (hERG) K^+^ current (I_Kr_) was inhibited by 4-ONE in hERG-overexpressing HEK cells. I_Kr_ was activated by depolarization from −60 to 30 mV, followed by repolarization of −40 mV evoked the maximum I_Kr_ activity, and the peak I_Kr_ was plotted to I–V relationship curve. (**B**) Slowly activating voltage-dependent K^+^ current (I_Ks_) was recorded from *KCNQ1/KCNE1*-overexpressing HEK cells. I_Ks_ was activated by depolarization from −40 to 60 mV. The maximum I_Ks_ was analyzed with I–V relationship curve. (**C**) L-type Ca^2+^ current (I_Ca,L_) was activated by applying from −40 to 60 mV of depolarization potentials from −50 mV of holding potential in guinea-pig ventricular myocyte (GPVM). The peak I_Ca,L_ was plotted to I–V relationship curve. (**D**) The decay of I_Ca,L_ was fitted using a double exponential equation. The slow component of time constant (τ_slow_) of I_Ca,L_ activated by 0 and 10 mV of depolarization potential was indicated.

The effects of 4-ONE on cardiac K^+^ channels were evaluated using the hERG and *KCNQ1/KCNE1* expressing HEK-293 cells. The acute treatment of 10 μM of 4-ONE reduced the peak amplitudes of I_Kr_ (hERG K^+^ current) and I_Ks_ (*KCNQ1/KCNE1* current) by 65% (Figure 4A; peak current density of 73.91 ± 7.55 and 25.93 ± 5.32 pA/pF at 20 mV for control and 4-ONE, respectively, *n* = 8) and 29%, respectively (Figure 4B; peak current density of 35.37 ± 8.95 and 25.66 ± 5.39 pA/pF at 40 mV for control and 4-ONE, respectively, *n* = 6). The cardiac I_Ca,L_ was recorded from GPVMs. The peak amplitude of I_Ca,L_ was decreased by 45% (Figure 4C; peak inward current at 0 mV of −4.30 ± 0.29 and −2.36 ± 0.36 pA/pF for control and 4-ONE, respectively, *n* = 5). It was notable that 4-ONE also slowed the inactivation of I_Ca,L_ (Figure 4D). When the inactivation phase of I_Ca,L_ was fit to double exponential function, the slow component of time constant (τ_slow_) became larger by 4-ONE at 0 and 10 mV (Figure 4D; τ_slow_ at 0 mV of 122.8 ± 11.68 and 145.9 ± 10.94 ms and τ_slow_ at 10 mV of 126.1 ± 9.92 and 144.3 ± 2.77 ms for control and 4-ONE, respectively, *n* = 7).

### 3.3. APD Prolongation and Increased Risk of Arrhythmia by 4-ONE

**Figure 5:**
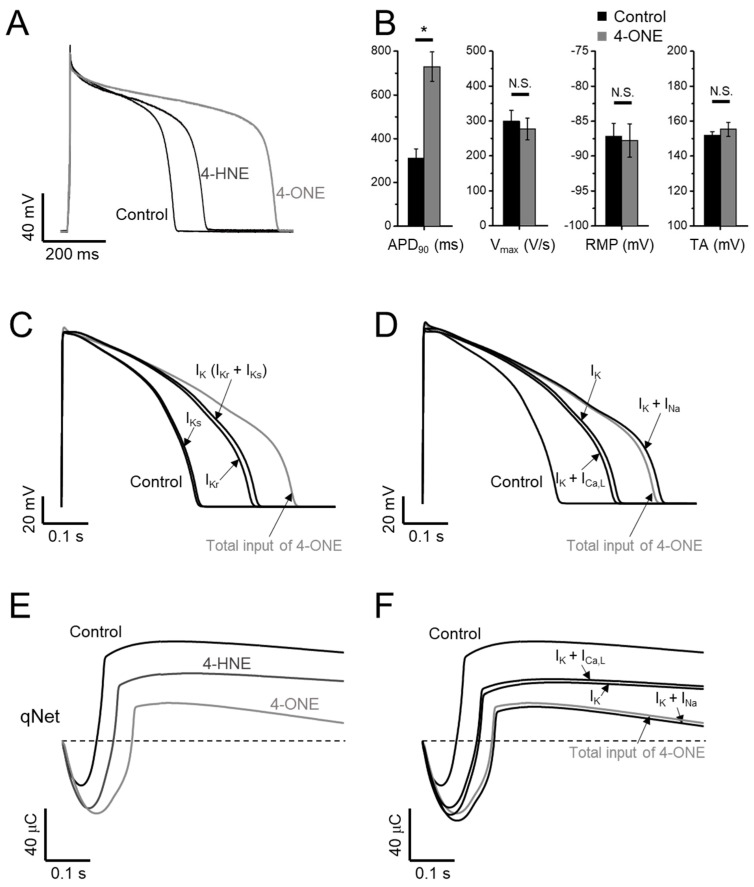
Effects of 4-ONE and 4-HNE on guinea-pig action potential (AP) and in silico AP. (**A**,**B**) Representative traces of AP show prolonged AP duration (APD) by 4-HNE (100 μM) and 4-ONE (10 μM) in GPVMs. (**B**) The APs were analyzed by APD at 90% repolarization (APD_90_), maximum overshoot velocity of AP (V_max_), resting membrane potential (RMP), and total amplitude (TA). (**C**–**F**) A CiPAORdv1.0 cell model was used for 4-ONE and 4-HNE simulation. (**C**) The contribution of APD prolongation simulated by I_K_ (I_Kr_ and I_Ks_) input by 4-ONE. (**D**) The contribution of APD prolongation simulated by I_Ca,L_ and I_Na_ (I_NaV_ and I_NaL_) added to I_K_ input. (**E**) qNet (net charge carried by total ionic currents) was calculated under 4-HNE and 4-ONE inputs. (**F**) The contribution of qNet simulated by I_Ca,L_ and I_Na_ added to I_K_ input. All the data were analyzed using paired *t*-tests, where a *p* < 0.05 was considered statistically significant (*).

The effects of 4-ONE on the cardiac AP were analyzed in GPVM under the current-clamp condition and triggered at 1 Hz. The bath application of 10 μM of 4-ONE markedly prolonged the APD (Figure 5A, B; APD_90_, 309.2 ± 44.50 and 729.4 ± 67.07 ms for control and 4-ONE, respectively, *n* = 10), which was more prominent than the effect of 100 μM of 4-HNE, as reported previously [13]. The maximum depolarization speed and total amplitude of APs were not affected by 4-ONE. In addition, the resting membrane potential of GPVMs was not changed (Figure 5B, right).

The CiPA, different from the conventional cardiotoxicity analysis investigating I_Kr_ only, covers the measurements of I_Kr_, I_Ca,L_, I_NaV_, and I_NaL_ for the analysis using the in silico model (CiPAORdv1.0: modified O’Hara–Rudy ventricular myocyte model). Using CiPAORdv1.0, we simulated the AP reflecting the electrophysiological changes induced by 4-ONE treatment (Figure 5C,D). For the calculation of the effects of 4-ONE, the relative conductance of I_Kr_ and I_Ks_ was decreased to 0.4 and 0.7, respectively. For I_Ca,L_, I_NaV_, and I_NaL_, in addition to the relative conductance, the changes of inactivation kinetics induced by 4-ONE were applied (Table 1). For 4-HNE simulation, the inputs with reduced I_Kr_ and I_Ks_ were applied according to our previous report [13]. The simulated APs revealed markedly prolonged APD by total input of 4-ONE. The decrease in I_Kr_ was more effective than that of I_Ks_ for the APD prolongation. However, it was notable that the modifications of both K^+^ currents (I_Kr_ and I_Ks_) were insufficient to simulate the change by 4-ONE (Figure 5C). The changes of inward currents (I_NaV_, I_NaL_, and I_Ca,L_) were additionally introduced. While the changes of I_Ca,L_ (slower inactivation and reduced conductance) had an insignificant effect, the increase in I_NaL_ showed a significant additional prolongation of APD (Figure 5D).

The risk of severe arrhythmia, such as Torsades de Pointes (TdP), is evaluated by a novel in silico biomarker, qNet (net charge carried by total ionic currents), proposed from CiPA [24,25]. The decrease in qNet by 10 μM of 4-ONE was more significant than that by 100 μM of 4-HNE (Figure 5E), indicating a higher risk of 4-ONE for arrhythmia induction. In addition, the sufficient reduction of qNet was observed by combining the changes of I_Kr_, I_Ks_, and I_Na_, but not by the simulation using the changes of I_Kr_, I_Ks_, and I_Ca,L_ (Figure 5F), which were consistent with the results of the stepwise simulation of APD change induced by 4-ONE.

## 4. Discussion

Our present study shows prominent cardiac APD prolongation by 4-ONE (10 μM) with multiple effects on the cardiac ion channels. We have previously reported that 100 μM of 4-HNE also induces APD prolongation with the inhibition of I_Kr_ [13]. In addition to the difference in the effective concentrations of the RCS, the APD prolongation and the risk of arrhythmia predicted by CiPA were commonly more prominent with 10 μM of 4-ONE than with 100 μM of 4-HNE (Figure 5). The genetic dysfunction or pharmacological inhibition of I_Kr_ has been regarded as one of the main mechanisms of APD prolongation and EAD. While sharing the inhibitory effect on I_Kr_ with 4-HNE, an additional intriguing finding was the augmentation of I_NaL_ by 4-ONE (Figure 2).

### 4.1. I_NaL_ and Inactivation of Na_V_1.5

The very rapid activation of hNa_V_1.5 is responsible for the fast activation wave and synchronous initiation of cardiac contraction. The inactivation process is also rapid, which prevents wasteful Na^+^ entry throughout the AP plateau in cardiomyocytes. However, cardiac I_NaV_ also shows residual flow during the sustained depolarization. Although I_NaL_ is relatively negligible to the fast component (0.1%–0.5% of peak I_NaV_), the continuous activity in the AP plateau could contribute to determining the shape and duration of the cardiac AP. Congenital gain-of-function mutations in *SCN5A* coding hNa_V_1.5 cause LQT-3. LQT-3 patients have a high risk not only for TdP but also for atrial fibrillation [14,15,16].

I_NaL_ is generally thought to be a persistent opening of the channels modulated either to slow the inactivation or to reopen over the voltage ranges between steady-state activation and inactivation curves, called a “window” potential. An enlargement of the window potential could be induced by the shift of activation or inactivation curves and has been reported as a mechanism of LQT-3 [14,28,29]. In our results, 4-ONE slightly shifted the inactivation curve to the left, implying a narrowed window potential at the relatively positive ranges (Figure 1D, right panel). Considering the voltage difference between the AP plateau (>0 mV, Figure 5) and the window potential under treatment with 4-ONE (below −40 mV, Figure 1E), it is unlikely that the current during the window period of AP could play a significant role for I_NaL_ induction [30].

More importantly, we found that the speed of hNa_V_1.5 inactivation was slowed by 4-ONE but not by 4-HNE (Figure 2). Cardiac I_NaV_ flows through a channel formed by the α-subunit encoded by *SCN5A*, which alone accounts for major features of I_NaV_ including the fast inactivation. A previous study suggested a structure responsible for the fast inactivation of I_NaV_ resides in IFM motif (isoleucine-phenylalanine-methionine) on the linker between the third and fourth repeat (DIII–DIV linker) as a “ball” or “lid” and on the bottom of the S4–S5 linker of each repeat (Figure 3E) [31]. In addition to the classical domain for fast inactivation, the perturbation of many locations can destabilize the inactivation and cause pathological I_NaL_ [31,32,33].

For the mechanisms of I_NaL_ by physiological PTM of hNa_V_1.5, CaMKII-dependent phosphorylation of Ser^571^ [34] and PKC-dependent phosphorylation of Ser^1503^ [35] have been reported. In addition, the nNOS (NOS1)-dependent S-nitrosylation was suggested, although the precise location of the candidate Cys has not been identified [36]. The non-congenital acquired increase in I_NaL_ is often observed in cardiomyocytes isolated from ischemic hearts and may be due to oxidative stress with increased ROS [37,38,39]. However, no previous study has paid attention to the modification of hNa_V_1.5 by 4-ONE that could be abundantly produced by ischemia/reperfusion conditions. In this regard, our present study might suggest a novel mechanism of I_NaL_ induction by ischemia/reperfusion-induced oxidative stress of the heart.

Through the MS/MS analysis, we could identify the binding sites of 4-ONE to hNa_V_1.5 (His^445^, His^472^, Lys^496^, and Arg^878^). Since the electrophysiological changes by 4-ONE was not reversed by washout with control solution, we carefully suggest that PTM sites revealed by the MS/MS analysis might be the candidate for the slowed inactivation and the increase in I_NaL_ (Figure 3). Although the modified residues are not equivalent to the reported mutations in the congenital LQT-3 patients [14,16,32], those sites are relatively close to the binding sites of a known I_NaL_ activator, veratridine (Figure 3E) [40,41]. The site-directed mutagenesis of hNa_V_1.5 and the electrophysiological investigation are requested to identify the actual roles of the modified residues in the I_NaL_ and the altered inactivation. Regretfully, we have not conducted the MS/MS analysis with hNa_V_1.5-HEK cells treated with 4-HNE. Since the treatment with 4-HNE did not induce the functional changes in I_NaV_ inactivation and I_NaL_, the comparative analysis might provide more specific information for the critical residue(s) of Na_V_1.5 modified by 4-ONE.

### 4.2. Pathophysiological Implication of 4-ONE and I_NaL_

4-ONE-mediated I_NaL_ induction might have a pathophysiological significance. Increased I_NaL_ in the heart can lead to arrhythmia by prolonging APD in a direct manner and by causing Ca^2+^ overload in an indirect manner. As for the former mechanism, the resurgent I_NaL_ at the repolarization phase of AP interferes with rapid repolarization and can cause EAD-associated arrhythmia. For the latter mechanism, the prolonged APD leads to Ca^2+^ overload by I_Ca,L_ and Na^+^–Ca^2+^ exchanger, triggering pathological Ca^2+^ release from intracellular Ca^2+^ storing organelles. The Ca^2+^ overload also causes diastolic dysfunction, increased wall stress, and ischemic risk [42]. In this regard, I_NaL_ has been suggested as an attractive therapeutic target to treat arrhythmia, heart failure, and angina. Ranolazine, the most selective clinical I_NaL_ inhibitor, has been used to suppress both arrhythmia events and angina [42,43]. In our result, 4-ONE-mediacted I_NaL_ was effectively reduced by 50 μM ranolazine (Figure 2B), further implying the pathophysiological role of 4-ONE in terms of the cardiac ischemia-associated arrhythmia.

### 4.3. Application of CiPA in Silico Model

To assess the arrhythmogenic risk of 4-ONE, we applied the CiPA in silico model. The inhibition of I_Kr_ alone could suggest a pathophysiological implication of 4-ONE. Interestingly, the qNet analysis and AP simulations revealed a higher risk of 4-ONE than of 4-HNE, which is due to the I_NaL_ induction. Such insight could not be obtained from the conventional cardiotoxicity test of the I_Kr_ analysis alone, which reflects the strength of CiPA that includes the integrative simulation of the multiple types of cardiac ion channels.

Another interesting feature of the present study was the slowed inactivation and the reduced peak amplitude of I_Ca,L_ by 4-ONE treatment, which was not observed in the previous study of 4-HNE [13]. However, according to the CiPA analysis, the enhanced persistent Ca^2+^ current modulated by the slowed I_Ca,L_ inactivation did not induce significant changes of the qNet and the simulated APD (Figure 5D,F), which appears to be due to the compensation by the decrease in peak current activation (Figure 4C).

## 5. Conclusions

Using electrophysiological investigation of cardiac ion channel currents, for the first time, we discovered the multichannel effects of 4-ONE, among which the inhibition of I_Kr_ and the induction of I_NaL_ were noteworthy, as confirmed by the qNet reduction indicating arrhythmogenic risk.

## Figures and Tables

**Table 1:** The input values for the simulation of AP using CiPAORdv1.0.

	Control	4-HNE	4-ONE
ths (I_Nav_)	$\frac{1.0}{0.009794e^{-\frac{v+17.95}{28.05}}+\;0.3343e^{\frac{v+5.730}{56.66}}}$		$\frac{1.0}{0.0036794e^{-\frac{v+17.95}{28.05}}+\;0.3343e^{\frac{v+5.730}{56.66}}}$
thL (I_NaL_)	200.0		400.0
tfcaf (I_Ca,L_)	$7.0+\frac{1.0}{0.04e^{-\frac{v-4.0}{7.0}}+\;0.04e^{\frac{v-4.0}{7.0}}}$		$7.0+\frac{1.0}{0.04e^{-\frac{v-10.0}{7.0}}+\;0.04e^{\frac{v-10.0}{7.0}}}$
tfcas (I_Ca,L_)	$100.0+\frac{1.0}{0.00012e^{-\frac{v}{3.0}}+\;0.00012e^{\frac{v}{7.0}}}$		$100.0+\frac{1.0}{0.00006e^{-\frac{v-10}{3.0}}+\;0.00006e^{\frac{v-10}{7.0}}}$
Conductance for I_Kr_	1.0	0.6	0.4
Conductance for I_Ks_	1.0	0.8	0.7
Conductance for I_Ca,L_	1.0	1.0	0.6

## Data Availability

Data are contained within the article.
